# Risk Factors for Coronary Artery Calcifications in Overweight or Obese Persons with Prediabetes: Can They Predict T2 Diabetes and Coronary Vascular Events?

**DOI:** 10.3390/jcm12123915

**Published:** 2023-06-08

**Authors:** Milica Marjanovic Petkovic, Miljanka Vuksanovic, Dragan Sagic, Ivana Radovic, Ivan Soldatovic, Teodora Beljic Zivkovic

**Affiliations:** 1 Division of Endocrinology Diabetes and Metabolic Disorders, Zvezdara University Medical Center, Medical Faculty, University of Belgrade, 11000 Belgrade, Serbia; 2 Cardiovascular Institute Dedinje, Medical Faculty, University of Belgrade, 11000 Belgrade, Serbia; 3 Blood and Transfusion Institute of Serbia, 11000 Belgrade, Serbia; ivanaradovic2@gmail.com; 4 Institute of Medical Statistics and Informatics, Medical Faculty, University of Belgrade, 11000 Belgrade, Serbia; soldatovic.ivan@gmail.com

**Keywords:** prediabetes, coronary artery calcifications, coronary vascular events

## Abstract

Background: It is difficult to predict the risk of developing atherosclerotic cardiovascular disease in subjects with prediabetes and obesity. The aim of this study was to assess risk factors for coronary artery calcifications (CACs) and the development of type 2 diabetes (T2D) and coronary vascular events (CVEs) after 7 years in 100 overweight or obese persons with prediabetes, according to the baseline coronary artery calcium score (CACS). Methods: Lipids, HbA1c, uric acid, and creatinine were assessed. Glucose, insulin, and c-peptide were determined during an oral glucose tolerance test. Multi-sliced computerized tomography with evaluation of CACS was performed. After 7 years, the subjects were assessed for T2D/CVE. Results: CACs were present in 59 subjects. No single biochemical marker could predict presence of a CAC. After 7 years, T2D developed in 55 subjects (61.8% initially had both IFG and IGT). A gain in weight was the only contributing factor for T2D. Nineteen subjects developed a CVE; increased initial clustering of HOMA-IR > 1.9, LDL > 2.6, and mmol/Land TGL > 1.7 mmol/L and higher CACS were present in that group. Conclusions: No risk factors for CACs could be identified. A gain in weight is associated with T2D development, as are higher CACS and clustering of high LDL+TGL+HOMA-IR with CVEs.

## 1. Introduction

Prediabetes is a metabolic disorder characterized by elevated fasting glucose levels of 5.6–6.9 mmol/L, by American Diabetes Association (ADA) criteria [1], or 6.1–6.9 mmol/L by the joint European (EASD and ESC) guidelines [2]. The inclusion of glycated haemoglobin A1c (HbA1c) helps in diagnosis [3]. People with prediabetes have an unpredictable prognosis. They may further develop type 2 diabetes mellitus (T2D), atherosclerotic cardiovascular disease (ASCVD), nonalcoholic hepatic steatosis, chronic kidney disease, or diabetic-related complications. Alternatively, they may lose weight through lifestyle changes, normalize their fasting glucose levels and other metabolic changes, and decrease the risk of chronic diseases. Projections for the evolution of T2D from prediabetes are alarming. If untreated, 37% of patients with prediabetes will progress to T2D in 4 years’ time [4].

Obesity is a multifactorial chronic disease, relapsing and progressive in its course, necessitating a team/patient–centered approach and medical treatment [5]. Body mass index (BMI) quantifies the extent of obesity. The overweight range is from 25 to <30 kg/m^2^. Obesity is present when the BMI is 30 kg/m^2^ or higher, with further divisions to: Class 1 (BMI 30 to <35 kg/m^2^), Class 2 (35 to <40 kg/m^2^), and Class 3, categorised as severe obesity, with a BMI of 40 kg/m^2^ and higher. Fat tissue, especially visceral fat, is disease-generating. Although BMI does not quantify body fat, it correlates with dual-energy X-ray absorptiometry, which directly measures all body fat compartments. Hence, BMI adversely correlates with various health outcomes and excess deaths [6]. Waist circumference (WC) is an indicator of abdominal body fat, which is associated with cardiometabolic disease and cardiovascular disease (CVD) mortality. Thus, several organizations recommend WC and BMI in clinical evaluations [7]. Visceral fat is responsible for systemic inflammation, as it releases inflammatory cytokines and chemokines into the vasculature that facilitate atherosclerosis [8]. Hyperglicemia is a recognized cardiovascular risk factor. Hence, obesity in prediabetes further adds to CVD morbidity and mortality through induction of insulin resistance, oxidative stress, endothelial dysfunction, and hypercoagulability. Early identification of both increased body weight and prediabetes can modify the risk of CVD development [9]. Systemic intervention with lifestyle changes, medical therapy, and surgical procedures can improve CVD outcomes [10,11].

Atherosclerotic cardiovascular disease is often seen in patients with prediabetes and represents an important source of morbidity and mortality. Patients with prediabetes and ASCVD have a 36% higher risk of all-cause mortality and a 15% higher risk of coronary heart disease compared to persons with normal glucose levels [12]. Prediabetes is often accompanied by obesity, hypertension, or dyslipidaemia. ADA guidelines recommend simultaneous modification of all existing risk factors for greater benefits [13]. Physicians are faced with the dilemma of how to quantify the risk of developing T2D and ASCVD in a patient with prediabetes. Family history is factored into the risk, which is understandable to the patient. The scoring systems, which include cardiovascular risk factors, are helpful; they include Systematic Coronary Risk Evaluation (SCORE) [14], SCORE2, and SCORE2-OP [15].

Several studies have shown that the coronary artery calcium score (CACS), measured by multi-detector CT, has a greater prognostic value than risk factor scoring systems [16]. As people with T2D have accelerated atherosclerosis, they have an elevated CAC score [17]. Patients with prediabetes have a 2.36 times greater chance of developing coronary artery calcifications [18]. It has also been observed that subjects with prediabetes have a faster progression of coronary artery calcifications compared to normoglycemic individuals [19]. Nevertheless, screening for coronary artery disease in asymptomatic T2D and, especially, overweight or obese patients with prediabetes remains controversial [20].

The aim of our study was to assess whether common biochemical analysis, easily performed in an outpatient clinic, can predict the presence of coronary artery calcifications in overweight or obese subjects with prediabetes. We also assessed the presence of T2D and coronary vascular disease after a period of 7 years, according to the baseline CACS severity of these individuals.

## 2. Materials and Methods

### 2.1. Study Population

One hundred consecutive subjects 45–65 years of age, diagnosed with prediabetes and obesity by their general practitioners, were included in the study. All were Caucasian and lived in Belgrade, Serbia. They had fasting blood glucose values of 5.6–6.9 mmol/L and glycated haemoglobin of A1C (HbA1c) 5.7–6.4%, from the local laboratories, thus confirming the diagnosis of prediabetes. Their body mass index (BMI) was in the range of overweight and obese (25.0–39.9 kg/m^2^). Regarding arterial hypertension, only patients who were well regulated on ACE inhibitors or beta-blockers in minimal doses were included. The subjects signed an informed consent which informed them of the planned oral glucose tolerance test, other blood samplings, body measurements, multi-slice computerized tomography scans for coronary artery calcium (CAC) evaluation, and a follow-up in 7–8 years.

### 2.2. Exclusion Criteria

The subjects with diabetes (HbA1c ≥ 6.5% and fasting blood glucose level ≥ 7 mmol/L), severe obesity (BMI ≥ 40 kg/m^2^), cardiovascular disease, non-regulated hypertension (systolic ≥ 150 mmHg, diastolic ≥ 90 mmHg, on an equivalent of no more than 2.5 mg of ramipril and/or 2.5 mg of bisoprolol), malignant neoplasm, chronic renal failure (CrCl for women ≤ 85 mL/min/1.73 m^2^, CrCl for men ≤ 75 mL/min/1.73 m^2^), active liver disease (except nonalcoholic fatty liver disease), hyperparathyroidism, and pregnancy were excluded.

### 2.3. Biochemical Data Collection and Measurements

The subjects were recruited from the endocrinology outpatient clinic of the Zvezdara University Medical Centre in Belgrade during the period of June 2013 to April 2015. They were referred to the endocrinologist because of prediabetes and/or obesity. Upon signing the informed consent form, they had a daily visit at the Centre for metabolic and demographic evaluation.

A daily visit was performed, after no caloric intake for at least 8 h. Body weight, height, and waist circumference were measured. Smoking habits, the presence of hypertension, present medication, and a family history of diabetes mellitus and coronary heart disease were noted. Blood samples were drawn for biochemical serum analysis of fasting plasma glucose, c-peptide, insulin, HbA1c, total serum cholesterol (TsC), high-density lipoprotein cholesterol (HDL-C), serum triglycerides (TGL), uric acid, and creatinine. HbA1c was performed by a turbidimetric inhibition immunoassay TINIA, TsC by an enzymatic method with cholesterol-oxidase, HDL-C by an enzymatic method with magnesium and dextran sulfate, TGL by an enzymatic method with lipase, glycerol-oxidase and PAP without glycerol correction, uric acid by an enzymatic method with uricase and a presence of peroxidase, and 4-aminophenazone and creatinine by the JAFFE method.

An oral glucose tolerance test (oGTT) with 75 g glucose was performed. In total, 75 g of glucose was dissolved into 300 mL of water, and the subjects drank it in a couple of minutes. Blood samples were again drawn for glucose, c-peptide, and insulin 2 h after the 75 g of oral glucose load. For reference ranges, we used fasting levels of insulin (6–27.0 mIU/L) and c-peptide (0.9–3.83 ng/mL), and insulin (16–166 mIU/L) and c-peptide (3–7.1 ng/mg) in the second hour of the oGTT [21]. The c-peptide and insulin measurements were performed with a biochemistry analyser, Cobas pro (Roche Diagnostics), using the chemiluminescent immunoassay (CLIA) technique at the Institute for Laboratory Diagnostics Konzilijum in Belgrade.

### 2.4. Clinical Data Calculations

Low-density lipoprotein cholesterol (LDL-C) was calculated automatically in the laboratory by the Friedewald formula: LDL-C (mmol/L) = TsC (mmol/L) − HDL − C (mmol/L) − TGL (mmol/L) divided by 2.2 [22].

The homeostasis model assessment for insulin resistance (HOMA-IR) was calculated according to the formula: HOMA-IR = fasting plasma glucose (mmol/L) × fasting serum insulin (mIU/L) divided by 22.5 [23]. The HOMA B cell function index (HOMA-B) was calculated as a product of 20 and (basal serum insulin levels (mIU/L), divided by plasma glucose (mmol/L)) and subtracted by 3.5 [24]. Creatinine clearance (CrCl) was estimated according to the Cockcroft–Gault formula: [(140-age (years)] × body weight (kg) × 1.23) divided by serum creatinine (µmol/L) [25].

### 2.5. Coronary Artery Calcification Evaluation

All the subjects were scheduled for multi-slice computerized tomography (MSCT) scans for coronary artery calcium score (CACS) evaluation. A GE Lightspeed 64-slice scan, without ECG gating and contrast injection, was performed on all the participants at the Institute for Cardiovascular Diseases Dedinje in Belgrade. As calcifications absorb more radiation than the surrounding soft tissue, calcifications appear white on MSCT scans. Attenuations and the area of all the coronary artery calcifications were measured, and the data were further processed by the cardiac scoring software Advantage Workstation AW 4.3. The images were evaluated by the same radiologist qualified in evaluating coronary artery calcifications. Individual scores for the four arteries (the right coronary artery, the left main coronary, the left anterior descending artery, and the left circumflex coronary artery) and the total coronary artery calcium score (CACS) in Agatson units (AU) were determined [26,27]. The following 5 CACS categories were distinguished: absence of CAC (CACS = 0 AU); mild CAC (CACS = 1–10 AU); moderate CAC (CACS = 11–100 AU); advanced CAC (CACS = 101–400 AU); and extensive CAC (CACS above 400 AU).

### 2.6. Follow-Up Outcomes

During the first four months of 2022, the subjects were contacted by phone once more. They were invited for an outpatient visit to evaluate the occurrence of T2D and coronary vascular events (CVE) consisting of: myocardial infarction (MI), angina pectoris (AP), and myocardial revascularization (implantation of a stent in coronary arteries or aortocoronary bypass). Those who could not come scanned and emailed or posted copies of their medical records.

The occurrence of T2D was assessed through medical records. If no medical records existed on type 2 diabetes, HbA1c and fasting glucose levels were tested at the Zvezdara University Medical Centre laboratory. A diagnosis of type 2 diabetes was established with fasting glucose levels of ≥7.0 mmol/L and HbA1c ≥ 6.5%. In all the patients, the BMI was calculated according to present weight and height.

### 2.7. Statistical Analysis

All the data were analysed using the statistical package SPSS (Statistical Package for the Social Sciences) v.20.0 (IBM Corp., Armonk, NY, USA). The results are presented as means ± standard deviation (SD) or median (25th–75th percentile) for continuous variables (age, BMI, lipid status, etc.) depending on the type of distribution. Normal distribution was tested using the Kolmogorov–Smirnov test and by the visual analysis of histograms of frequencies and Q-Q plots. For categorical variables (sex, smoking, etc.), the results are presented as count (n) and percentage (%). Groups with different stages of coronary artery calcifications were compared using parametric (Student’s *t*-test) and nonparametric (Chi-square, Fisher’s exact, and Mann–Whitney U) tests, depending on the type of variable and its distribution.

The difference in the prevalence of T2D and CVE among various groups after a seven-year follow-up period was assessed by nonparametric (Chi-square, Fisher’s exact test, and linear by linear association) tests. For continuous variables, the difference between the groups with and without the outcome of interest was assessed by parametric (Student’s *t*) and nonparametric (Mann–Whitney U) tests, depending on the type of distribution. Normal distribution was tested using the Shapiro–Wilk test and by the visual analysis of histograms of frequencies and Q-Q plots. All *p*-values less than 0.05 were considered significant.

## 3. Results

### 3.1. Clinical Characteristics and Biochemical Results of Subjects with or without Coronary Artery Calcifications

The study involved 100 subjects, 29 of whom were male. Their demographics and clinical and biochemical results are shown in Table 1. The average age of the subjects was 56.76 ± 6.86 years. All the female participants were postmenopausal. The patients had an average BMI of 29.23 ± 4.50 kg/m^2^ and a waist circumference of 97.05 ± 11.93 cm. Arterial hypertension was present in 62 patients. Smoking is still popular in Belgrade; hence, 42 of the 100 subjects were current smokers. Family history for T2D (presence of diabetes in parents, siblings, and grandparents) was positive in 83 patients.

The average value of triglycerides for all 100 subjects was above the reference range (≤1.7 mmol/L). The same holds true, but to a lesser degree, for total cholesterol. LDL cholesterol was higher than recommended by the 2019 ESC/EAS Guidelines [2]. The average values of HDL cholesterol, uric acid, creatinine, and creatinine clearance were in the normal range in all the overweight or obese subjects with prediabetes.

All the subjects underwent the MSCT coronary artery calcium scans. Coronary artery calcium was absent (CACS = 0 AU) in 41 of the 100 subjects. It was positive in 59 subjects. No difference was observed in the presence of CAC according to the three classes of BMI. Of the 14 subjects with normal BMI, 9 had CAC and 5 did not. Forty-four subjects were overweight; CAC was present in 25 and absent in 19 subjects. Obesity was present in 42 subjects, 25 of whom had CAC, while it was absent in 17.

Mild CAC (CACS = 1–10 AU) was initially present in 10 subjects. Moderate CAC (CACS = 11–100 AU) was present in 24 subjects, while 17 subjects had initially advanced CAC (CACS = 101–400 AU) and 8 had extensive CAC (CACS above 400 AU). No difference in gender, age, BMI, waist circumference, presence of hypertension, or family history was observed between the 41 subjects with absent and 59 with present coronary artery calcifications. Smoking was more frequent among subjects with positive coronary artery calcium, approaching statistical significance (Table 1).

### 3.2. Parameters Indicating Glucose Metabolism

More than half of the overweight or obese subjects with prediabetes (66/100) had both impaired fasting glucose and glucose intolerance. Impaired fasting glucose was present in 28 subjects, while 6 subjects had only impaired glucose tolerance. This was independent of the presence or absence of coronary artery calcium (Table 2).

The average HbA1c and glucose levels during oGTT were in accordance with the ADA 2023 criteria that define prediabetes [1]. The fasting levels of insulin and C-peptide were in the normal range. The insulin and c-peptide in the second hour of the oGTT did not differ between the groups with negative and positive coronary artery calcium.

The median value of the HOMA-IR index was high in all 100 subjects with prediabetes and did not differ between the groups. No difference was observed in the HOMA-B index among subjects with absent or present coronary artery calcium. In 34 subjects, the HOMA-IR was higher than 1.9. It was accompanied by LDL-C and TGL values above 2.6 mmol/L and 1.7 mmol/L, respectively. However, no difference in the distribution of this cluster (HOMA-IR higher than 1.9, LDL higher than 2.6 mmol/L and TGL higher than 1.7 mmol/L) regarding the presence of CAC was observed (Table 2).

### 3.3. Follow-Up Outcomes

In 2022, we managed to contact 77 subjects once again. They came for an outpatient visit. The remaining 23 subjects sent copies of their medical records. During the seven-year period, the patients were monitored by the general practitioners. Some were referred to the endocrinologist or cardiologist. Weight measurement with BMI calculation on follow-up revealed that, after 7 to 8 eight years, the initial 14 subjects with normal BMI no longer had an increase in BMI. Eleven of them became overweight, and 3 became obese. The 3 that were initially of normal weight and subsequently became obese developed T2D and had a CVE. Of the 11 that became overweight, 6 developed T2D, and none a CVE. Of the initial 44 overweight subjects, 4 became obese. Initially obese subjects remained obese. Of the 4 who were initially overweight and then became obese, 2 developed T2D and 1 a CVE. The change in the number of overweight and obese subjects with prediabetes, after a seven-year period when some developed T2D and some had CVE, is shown in Table 3.

Regarding outcomes of interest, T2D developed in 55% of the now overweight or obese subjects with prediabetes. Both IFG and IGT were initially present in the majority of the subjects who later developed T2D (61.8%). About one-third had only IFG (32.7%), and only 5.5% had IGT. When the initial glycaemic disarrangement was compared to those who did not develop T2D, the difference was not significant (*p* = 0.527). We did not find a difference in gender, age, initial BMI, waist circumference, hypertension, smoking, family history, or initial lipid parameters between those who developed T2D and those who did not (Table 4). Additionally, initial glucose, insulin, and c-peptide levels during the oral glucose tolerance test, HOMA-IR, HOMA-B, and clustering of high HOMA-IR with high levels of LDL-C and TGL could not predict future development of T2D. The initial coronary artery calcium was not different in patients that developed T2D from those who did not. However, the only difference that was observed between subjects who developed and those who did not develop T2D was a gain in weight, which led to a significant rise in BMI (Table 4). Lipid status was not evaluated after the follow-up period, because the subjects were predominantly on statin therapy.

**Figure 1:**
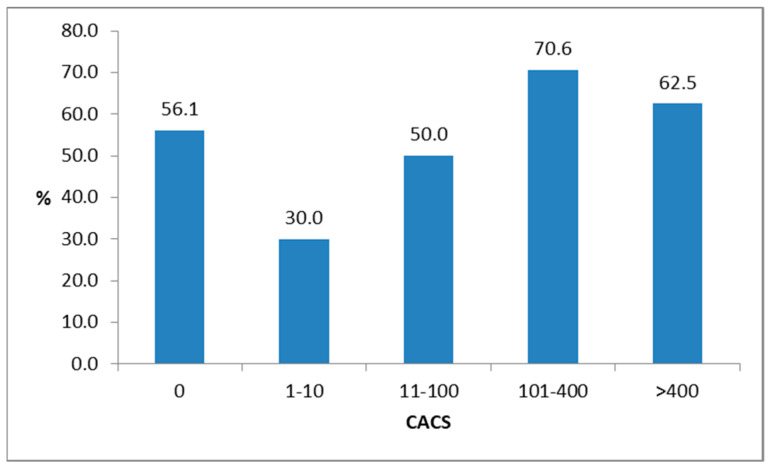
Incidence of T2D in % after a seven-year period in subjects with prediabetes and different coronary artery calcium scores (CACSs).

We then analysed the incidence of T2D after at least seven years among the five original categories of CACS. No association was observed between the occurrence of T2D and the initial coronary artery calcium score (*p* = 0.427). Type 2 diabetes developed in 23 of the 41 subjects (56.1%) with initially no CAC. It occurred in 3 of the 10 subjects with mild CAC (30%) and in 12 of the 24 with moderate CAC (50%). Type 2 diabetes was most common among those with advanced CAC (12 out of 17 cases; 70.6%). It was also common in the group with extensive CAC, as five of the eight subjects (62.5%) developed T2D (Figure 1).

Nineteen subjects suffered from one or more CVEs. The most frequently diagnosed coronary vascular event was angina pectoris. The diagnosis of angina pectoris was made after a positive cardiac exercise stress test. It was observed in 11 subjects. They were treated medically. Three subjects suffered a myocardial infarction (1 had ST elevation (STEMI), and 2 were without ST elevation (NSTEMI)). The patient with a STEMI myocardial infarction had three stent placements. One patient with a NSTEMI had one stent placement, while the other was treated medically. Two subjects were diagnosed with chronic heart failure with a preserved ejection fraction of the left ventricle. Two subjects had stent placement on elective coronarography, while one had aortocoronary bypass surgery, after elective coronarography. Of these 19 subjects, 15 also developed T2D.

The majority of the subjects (63.2%) who suffered a coronary vascular event had both IFG and IGT initially. The remaining 36.8.7% had isolated IFG, and none had isolated IGT. The difference between those who did not develop a coronary vascular event was not significant (*p* = 0.431). Subjects who were active smokers were more prevalent in the group with CVE (73.7% vs. 34.6%, *p* = 0.002). We did not find a difference in gender, age, initial BMI, change in BMI during the follow-up period, initial waist circumference, hypertension, family history, initial lipid parameters, initial glucose, insulin and c-peptide levels during the oral glucose tolerance test, HOMA-IR, or HOMA-B between those who developed a coronary vascular event and those who did not. Nevertheless, subjects with coronary vascular events more frequently had an initial cluster of high LDL-C, TGL and HOMA-IR (57.9% vs. 27.5%, *p* = 0.015). Although 14 of the 19 patients who had a coronary vascular event had positive initial coronary artery calcium, this was not different from the initial CAC of those who did not develop a CVE (Table 5).

**Figure 2:**
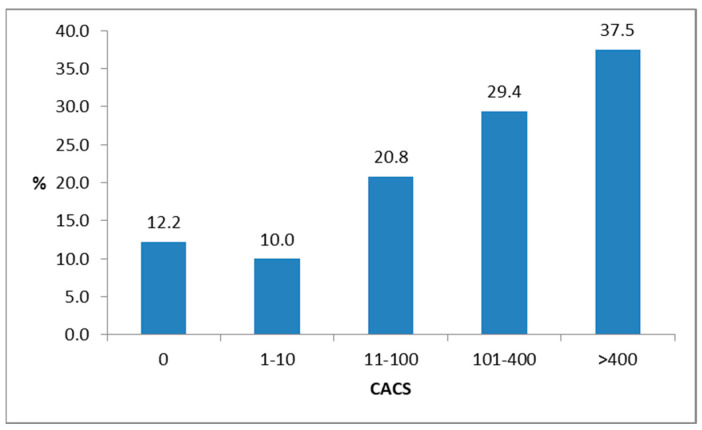
Incidence of coronary vascular events in % after a seven-year period in subjects with prediabetes and initially different coronary artery calcium scores (CACS).

The incidence of CVE in five CACS categories can be observed in Figure 2. Five of forty-one (12.2%) subjects who had no CAC suffered a coronary vascular event. Of the ten subjects with mild CAC, one (10.0%) had a CVE. The group with moderate CAC was the largest, with 24 subjects. Of them, five (20.8%) had a CVE. Incidences of CVE among subjects with advanced and extensive CAC were 5 of 17 (29.4%) and 3 of 8 (37.5%), respectively. This difference was statistically significant, and an increasing trend was observed (*p* = 0.041).

## 4. Discussion

Our study was performed with the aim of providing an answer as to whether routine biochemical testing, combined with an evaluation of the coronary artery calcium score, could predict the development of T2D and coronary vascular disease in overweight or obese subjects with prediabetes. We observed that our participants had higher-than-recommended levels of triglycerides and LDL cholesterol. However, no difference in biochemical markers, parameters indicating glucose metabolism, or clinical characteristics, except for smoking, was observed between subjects who had coronary artery calcifications and those who did not.

After a period of 7 years, T2D developed in 55% of our overweight or obese subjects with prediabetes. A gain in weight was the only identifiable factor contributing to T2D development. No association was observed between the occurrence of T2D and the initial coronary artery calcium score. Nineteen subjects suffered from one or more coronary vascular events. An initial cluster of high LDL-C, TGL, and HOMA-IR was significantly more frequent in these subjects, as was active smoking (73.7%, *p* = 0.002). An increasing trend was observed between the severity of the initial CACS and the occurrence of coronary vascular events. Other authors also reported a positive association between smoking and CACS in patients with prediabetes and diabetes [28,29].

It is unclear whether impaired fasting glucose, per se, influences the development of coronary artery calcifications. Eun et al. suggested that IFG, especially fasting plasma glucose ≥6.1 mmol/L, may be an independent risk factor for the development of CAC [28]. A large Korean cohort study showed an increased risk of CACS > 100 among patients with prediabetes, the prevalence being 12–31% higher than in subjects with normal fasting glucose. CAC progression was also more prominent in patients with prediabetes [30]. There is, however, diversity of opinion on this matter. Liu et al. found that in the Chinese population, subjects with metabolic syndrome had a significantly higher risk of cardiovascular disease and ischemic stroke regardless of their glycemic status. They stated that in the absence of metabolic syndrome, IFG is associated with a higher hazard ratio for coronary heart disease and T2D with ischemic stroke [31]. In 3054, Framingham Heart Study participants without T2D or CAC showed an association with obesity but not with IFG [32]. Won et al. concluded that prediabetes was not a risk factor for CAC progression [33]. Some authors found an association between IFG and a higher prevalence of CAC only in men [34,35]. These differences in findings can in part be explained by the fact that the authors used different lower boundaries for IFG, ranging from 5.6 mmol/L to 6.1 mmol/L. In our study, we used the lower threshold for IFG and thus included subjects whose glucose metabolism was impaired to a lesser degree. This might have resulted in lower oxidative stress, inflammation, and endothelial dysfunction, important in the development of coronary artery calcifications. Subjects with absent CAC had lower average values of fasting glucose, but the difference was not significant.

In our group of subjects, the average values of LDL cholesterol, total serum cholesterol, and triglycerides were elevated. This is a common finding in patients with prediabetes [36]. In previous studies, different biochemical markers were identified as risk factors for CAC. Eun et al. suggested that elevated LDL-C and triglycerides are significant predictive factors for CAC in patients with IFG [28]. Scicali et al. reported that lipid levels were not associated with CAC, but higher HbA1c levels were [37]. We found no significant difference in lipid parameters and HbA1c between subjects with present and absent CAC. Likewise, we observed no significant difference in C-peptide and HOMA indexes. This absence of difference may be due to a relatively small sample size. Fakhrzadeh et al. reported a more than a 77.8% increase in the risk of CACS ≥ 10 in nondiabetics for every one-unit increase in HOMA-IR [38]. De Leon et al. reported increased risks for coronary artery disease and MI in nondiabetics with initial insulin resistance and elevated C-peptide but normal fasting glucose [39].

CACS measurement by CT scan enables the direct assessment of a patient’s coronary artery atherosclerotic plaque burden. Multiple studies report the association of CACS with traditional risk factors for atherosclerosis (age, sex, race, obesity, hypertension, T2D, and family history of coronary heart disease) both in patients with and without T2D [29,40,41]. This association is by no means definite. Coronary artery calcifications have been found in young people or those with one or no risk factors. In the elderly, or people with multiple risk factors, CAC may be absent. Lei et al. state that only the clustering of three or more risk factors may predict higher CACS. However, 20% of T2D patients with high risk factors had a CACS of zero [29]. In our study, we found a clustering of high-LDL cholesterol, high triglycerides, and HOMA IR higher than 1.9. Subjects with this cluster did not have a more prevalent CAC. However, this cluster was more prevalent in subjects who suffered a coronary vascular event during the 7 years of follow-up (57.9% *p* = 0.015).

This study showed a greater increase in BMI in subjects who developed T2D (*p* = 0.024). This observation is in accordance with other studies which demonstrated that lifestyle intervention aimed at reducing BMI and increasing physical activity could reduce the incidence of T2D [42].

CACS has been shown to be superior in assessing the risk of cardiac events and all-cause mortality in the asymptomatic general population [43,44,45]. The same holds true for patients with T2D. It has been observed that patients with CACS > 400 have a significantly higher hazard ratio for coronary revascularization (10.83), coronary heart disease (10.5), cardiac mortality (8.67), and all-cause mortality (2.08) when compared with patients with CACS = 0 [22]. Our study also showed that overweight or obese subjects with prediabetes have a significant (*p* = 0.041) rise in the incidence of coronary vascular events with increasing values of CACS.

Among patients who suffered a CVE were also those with a CACS of zero, but in a smaller proportion (26.3%). This can be explained by the fact that certain forms of coronary disease, such as “soft plaque” atherosclerosis, escape detection during CT scans. Cen et al. showed that patients with IGT had more soft plaque, eccentric plaque, and positive remodelling but less calcification with a larger burden and area of the plaque. This puts the patients at greater risk of having vulnerable plaque that can easily rupture, resulting in acute coronary syndrome [46]. Shemes et al. came to a similar conclusion, that extensive CAC was associated with chronic coronary events and that an absence or mild to moderate CAC was associated with acute events [47].

The main limitation of this study was the small number of subjects recruited. The reason for this was the low referral of these patients to endocrinologists, as they are monitored at the primary health care level. As a consequence, the differences between the groups were too small to be noted as statistically significant. Our plan for the future is to enlist more participants so that we will be able to detect subtle differences in biochemical parameters, which can be used to screen individuals at higher risk of development of CAC at the primary health care level. Another limitation is that we did not analyse hs-CRP, which could have given information on systemic inflammation and its role in the formation of coronary artery calcifications.

## 5. Conclusions

We were not able to detect a single biochemical marker, easily assessed in an outpatient setting, that could predict the presence of coronary artery calcifications in overweight or obese subjects with prediabetes. After a period of 7 years, T2 diabetes developed in 55% of our participants, the gain in weight being the only contributing factor. The incidence of coronary vascular events increased with the severity of coronary artery calcifications. An initial cluster of high LDL-C, TGL, and HOMA-IR was significantly more frequent in subjects who eventually developed a coronary vascular event.

## Figures and Tables

**Table 1:** Demographics and clinical and biochemical results of subjects with or without coronary artery calcifications.

	Total	CAC		*p*
		0	1+	
	N = 100	N = 41	N = 59	
Male gender	29 (29.0%)	10 (24.4%)	19 (32.2%)	0.397
Age (years)	56.76 ± 6.86	55.68 ± 6.28	57.51 ± 7.19	0.192
BMI (kg/m^2^)	29.23 ± 4.50	29.05 ± 4.33	29.35 ± 4.66	0.742
WC (cm)	97.05 ± 11.93	96.27 ± 11.50	97.59 ± 12.28	0.587
HTA	62 (62.0%)	24 (58.5%)	38 (64.4%)	0.552
Smoking	42 (42.0%)	13 (31.7%)	29 (49.2%)	0.082
FHT2D	83 (83.0%)	34 (82.9%)	49 (83.1%)	0.987
TsC (mmol/L)	5.61 ± 1.01	5.53 ± 0.88	5.66 ± 1.09	0.518
HDL-C (mmol/L)	1.15 ± 0.35	1.11 ± 0.33	1.17 ± 0.36	0.388
LDL-C (mmol/L)	3.37 ± 0.95	3.34 ± 0.85	3.39 ± 1.01	0.803
TGL (mmol/L)	2.47 ± 0.93	2.31 ± 0.95	2.58 ± 0.90	0.158
Uric acid (mmol/L)	318.23 ± 67.34	310.27 ± 78.89	323.76 ± 58.06	0.354
Creat. (µmol/L)	78.91 ± 13.28	78.56 ± 13.3	79.15 ± 13.37	0.828
CrCl	101.56 ± 26.8	99.2 ± 20.87	103.2 ± 30.31	0.465

BMI—body mass index, WC—waist circumference, HTA—hypertension, FHT2D—family history of type 2 diabetes, TsC—total serum cholesterol, HDL-C—high-density lipoprotein cholesterol, LDL-C—low-density lipoprotein cholesterol, Creat.—creatinine, CrCl—creatinine clearance, CAC—coronary artery calcifications.

**Table 2:** Biochemical parameters indicating glucose metabolism in subjects with or without coronary artery calcifications.

	Total	CAC		*p*
		0	1+	
	N = 100	N = 41	N = 59	
HbA1c (%)	5.9 ± 0.37	5.94 ± 0.39	5.87 ± 0.35	0.343
IFG	28 (28.0%)	9 (22.0%)	19 (32.2%)	0.296
IGT	6 (6.0%)	4 (9.8%)	2 (3.4%)	
IFG + IGT	66 (66.0%)	28 (68.3%)	38 (64.4%)	
Glucose 0 min (mmol/L)	6.41 ± 0.82	6.36 ± 0.9	6.45 ± 0.76	0.608
Glucose 120 min (mmol/L)	8.61 ± 1.8	8.64 ± 1.82	8.59 ± 1.80	0.892
Insulin 0 min (mU/L)	6.70 (3.75–10.95)	5.80 (3.70–11.40)	6.70(3.80–10.70)	0.925
Insulin 120 min (mU/L)	42.30 (18.15–62.80)	41.20 (19.30–58.60)	42.80 (12.10–71.90)	0.804
C-peptide 0 min (ng/mL)	2.25 (1.36–3.13)	2.18 (1.45–3.23)	2.28(1.10–3.03)	0.695
C-peptide 120 min (ng/mL)	8.27 (4.47–11.13)	8.36 (6.12–11.14)	7.94 (3.48–10.94)	0.207
HOMA-IR	1.87 (1.03–3.09)	1.79 (0.94–3.12)	1.91 (1.08–3.05)	0.825
HOMA-B	52.22 (25.77–75.84)	55.71 (25.94–73.55)	49.29 (22.93–82.94)	0.710
Cluster of high LDL-C, TGL and HOMA-IR	34 (34%)	12 (29.3%)	22 (37.3%)	0.405

HbA1c—glycated haemoglobin A1c, IFG—impaired fasting glucose, IGT—impaired glucose tolerance, HOMA-IR—the homeostasis model assessment-insulin resistance, HOMA-B—the homeostasis model assessment-beta cell function, CAC—coronary artery calcifications.

**Table 3:** Initial BMI categories and BMI categories after a seven-year period in groups that developed/did not develope T2 diabetes and coronary vascular events (CVE).

Initial Categories of BMI	Numbers of Subjects			
Normal weight	14 (14.0%)			
Overweight	44 (44.0%)			
Obese	42(42.0%)			
		T2D after 7 years N = 55	No T2D after 7 years N = 45	*p*
Categories of BMI after 7 years				
Overweight	51	25(45.4%)	26(57.8%)	0.220
Obese	49	30(54.5%)	19 (42.3%)	
		CVE after 7 years N = 19	No CVE after 7 years N = 81	
Overweight	51	8 (42.1%)	43 (53.1%)	0.389
Obese	49	11 (57.9%)	38 (46.9%)	

BMI—body mass index, T2D—type 2 diabetes mellitus, CVE—coronary vascular events.

**Table 4:** Characteristics of subjects who developed type 2 diabetes mellitus after a seven-year period, compared to those who did not develop type 2 diabetes.

	T2D after 7 Years N = 55	No T2D after 7 Years N = 45	*p*
Gender			
male	16 (29.1%)	13 (28.9%)	0.982
female	39 (70.9%)	32 (71.1%)	
Age (years)	56.51 ± 7.07	57.07 ± 6.65	0.688
BMI (kg/m^2^)	29.24 ± 4.67	29.22 ± 4.34	0.987
WC (cm)	97.29 ± 10.29	96.76 ± 13.78	0.830
HTA			
yes	33 (60.0%)	29 (64.4%)	0.649
no	22 (40.0%)	16 (35.6%)	
Smoking			
yes	27 (49.1%)	15 (33.3%)	0.112
no	28 (50.9%)	30 (66.7%)	
FHT2D			
yes	48 (87.3%)	35 (77.8%)	0.209
no	7 (12.7%)	10 (22.2%)	
TsC (mmol/L)	5.57 ± 0.98	5.65 ± 1.05	0.728
HDL-C (mmol/L)	1.13 ± 0.31	1.17 ± 0.39	0.595
LDL-C (mmol/L)	3.32 ± 0.85	3.43 ± 1.05	0.577
TGL (mmol/L)	2.49 ± 0.9	2.45 ± 0.97	0.842
Uric acid (mmol/L)	306.64 ± 67.66	332.4 ± 64.89	0.057
Creat. (µmol/L)	79.2 ± 13.5	78.56 ± 13.14	0.811
CrCl	102.2 ± 23.21	100.78 ± 30.89	0.793
HbA1c (%)	5.89 ± 0.34	5.9 ± 0.4	0.856
OGTT results			
IFG	18 (32.7%)	10 (22.2%)	0.527
IGT	3 (5.5%)	3 (6.7%)	
IFG + IGT	34 (61.8%)	32(71.1%)	
Glucose 0 min (mmol/L)	6.54 ± 0.71	6.26 ± 0.92	0.080
Glucose 120 min (mmol/L)	8.51 ± 1.92	8.74 ± 1.65	0.523
Insulin 0 min (mU/L)	6.6 (3.8–11.2)	6.7 (3.7–10.69)	0.956
Insulin 120 min (mU/L)	40.3 (17–57.1)	44.7 (18.8–95.98)	0.153
C-peptide 0 min (ng/mL)	2.18 (1.20–3.15)	2.25 (1.44–3.02)	0.798
C-peptide 120 min (ng/mL)	7.4 (4.19–9.9)	8.4 (4.62–13.23)	0.197
HOMA-IR	1.91 (1.1–3.12)	1.84 (1–2.9)	0.703
HOMA-B	49.29 (21.11–73.08)	53.33 (29.44–89.71)	0.404
High LDL-C, TGL and HOMA-IR			
yes	21 (38.2%)	13 (28.9%)	0.329
no	34 (61.8%)	32 (71.1%)	
CAC			
0	23 (41.8%)	18 (40.0%)	0.854
1+	32 (58.2%)	27 (60.0%)	
Change in BMI after follow-up (kg/m^2^)	2.15 ± 0.78	1.83 ± 0.79	0.049

HTA—hypertension, IFG—impaired fasting glucose, IGT—impaired glucose tolerance, FHT2D—family history of type 2 diabetes, T2D—type 2 diabetes, CAC—coronary artery calcifications.

**Table 5:** Characteristics of subjects who developed a coronary vascular event after a seven-year period compared to those who did not.

	CVE after 7 Years N = 19	No CVE after 7 Years N = 81	*p*
Gender			
male	7 (36.8%)	22 (27.2%)	0.403
female	12 (63.2%)	59 (72.8%)	
Age (years)	58.47 ± 6.68	56.36 ± 6.88	0.228
BMI (kg/m^2^)	29.06 ± 4.35	29.27 ± 4.56	0.854
WC (cm)	99.16 ± 10.63	96.56 ± 12.22	0.395
HTA			
yes	14 (73.7%)	48 (59.3%)	0.244
no	5 (26.3%)	33 (40.7%)	
Smoking			
yes	14 (73.7%)	28 (34.6%)	0.002
no	5 (26.3%)	53 (65.4%)	
FHT2D			
yes	17 (89.5%)	66 (81.5%)	0.516
no	2 (10.5%)	15 (18.5%)	
TsC (mmol/L)	5.35 ± 0.86	5.67 ± 1.03	0.214
HDL-C (mmol/L)	1.16 ± 0.38	1.14 ± 0.34	0.868
LDL-C (mmol/L)	3.22 ± 0.63	3.4 ± 1.01	0.333
TGL (mmol/L)	2.72 ± 0.95	2.41 ± 0.92	0.187
Uric acid (mmol/L)	336.26 ± 78.34	314 ± 64.31	0.196
Creat. (µmol/L)	83.26 ± 17.59	77.89 ± 11.96	0.113
CrCl	98.21 ± 26.71	102.35 ± 26.93	0.548
HbA1c (%)	5.84 ± 0.37	5.91 ± 0.37	0.430
OGTT results			
IFG	7 (36.8%)	21 (25.9%)	0.431
IGT	0 (0.0%)	6 (7.4%)	
IFG + IGT	12 (63.2%)	54 (66.7%)	
Glucose 0 min (mmol/L)	6.61 ± 0.52	6.37 ± 0.87	0.120
Glucose 120 min (mmol/L)	8.41 ± 1.9	8.66 ± 1.79	0.590
Insulin 0 min (mU/L)	6.9 (4–11.2)	6.7 (3.7–10.69)	0.641
Insulin 120 min (mU/L)	42.8 (11.9–57.1)	42.2 (18.8–69.7)	0.592
C-peptide 0 min (ng/mL)	2.37 (1.64–3.51)	2.18 (1.18–3.03)	0.263
C-peptide 120 min (ng/mL)	8.38 (6.22–9.9)	7.71 (4.33–11.14)	0.830
HOMA-IR	2.02 (1.12–2.99)	1.83 (1–3.17)	0.474
HOMA-B	49.29 (19.05–88.46)	53.33 (25.94–71.43)	0.982
High LDL-C, TGL and HOMA-IR			
yes	11 (57.9%)	23 (28.5%)	0.015
no	8 (42.1%)	58 (71.6%)	
CACS			
0	5 (26.3%)	36 (44.4%)	0.148
1+	14 (73.7%)	45 (55.6%)	
Change in BMI after follow-up (kg/m^2^)	2.11 ± 0.77	1.98 ± 0.80	0.548

HTA—hypertension, IFG—impaired fasting glucose, IGT—impaired glucose tolerance, FHT2D—family history of type 2 diabetes, T2D—type 2 diabetes, CVE—coronary vascular event, CACS—coronary artery calcifications.

## Data Availability

The data will be shared on reasonable request to the corresponding author (with additional anonymisation to avoid patient identification).
